# A Bibliometric Analysis and Systematic Review of Research Advances in In Situ Gel Drug Delivery Systems from 2003 to 2023

**DOI:** 10.3390/pharmaceutics17040451

**Published:** 2025-04-01

**Authors:** Huanyu Zhou, Mingqing Yuan, Tao Zhang

**Affiliations:** Guangxi Key Laboratory of Special Biomedicine, Medical School of Guangxi University, Nanning 530004, China; 2228391066@st.gxu.edu.cn (H.Z.); zhangtao@fuanpharm.com (T.Z.)

**Keywords:** in situ gel, drug delivery system, in situ gel formulation, hydrogel, bibliometrics, VOSviewer

## Abstract

**Objective**: We aimed analyze research trends in in situ gel drug delivery systems. **Methods**: Studies from 2003 to 2023 were systematically obtained from the Web of Science database and analyzed using VOSviewer software to evaluate publication trends, citation patterns, and collaborative networks. **Results**: A total of 990 articles were reviewed. There has been a significant increase in publications since 2019, with the highest number of publications occurring in 2023. China was the leading country in terms of publication output. Cairo University and King Abdulaziz University were identified as the top contributing institutions. Key researchers included Zhao, Xia, Hosny, and Kim. The research primarily focused on developing new formulations, optimizing materials (e.g., biocompatible and biodegradable materials), and exploring clinical applications such as nasal–brain delivery for Alzheimer’s treatment. **Conclusions**: In situ gel systems have gained widespread use in clinical practice due to their ability to provide prolonged drug release and enhance patient compliance. This area remains crucial for future research, particularly in formulation design and administration methods.

## 1. Introduction

With the advancement of medical technology and scientific progress, gel materials synthesized under mild conditions are gaining prominence. Notably, in situ gels hold considerable promise within the realm of biomedicine. The in situ gel drug delivery system boasts exceptional tissue compatibility, superior injectability, prolonged retention, and a high drug-loading capacity. It is capable of providing sustained drug release while exhibiting minimal susceptibility to the body’s internal environment. As a result, it has garnered significant attention in the domains of pharmaceutics and biotechnology [1]. In recent years, as shown in Table 1,the innovation of novel dosage forms for in situ gel delivery systems has garnered substantial attention within the biomedical field. Newly developed injectable in situ gels exhibit low-viscosity aqueous characteristics in vitro. However, upon administration to the targeted site, they undergo a sol–gel transition, transforming into solid or semi-solid hydrogels. This transformation occurs through mechanisms of physical and/or chemical crosslinking, enabling effective and localized drug delivery [2]. Due to its unique phase transition properties, it can prolong the drug’s retention time at the site and form a high local drug concentration. Therefore, in situ gel drug delivery systems often possess multiple targeting effects. In situ gels have been widely studied and applied in drug delivery, three-dimensional cell culture, injectable tissue engineering, surgical adhesives, tissue sealants, anti-adhesion coatings, etc. [3].

With the ongoing exploration of novel technologies within the realm of pharmaceutical formulations and the comprehensive study of biodegradable polymer materials, an array of innovative dosage forms has been developed. In situ gels, as a pioneering injectable long-acting drug delivery system, have captured significant attention from pharmaceutical research enterprises. This unique dosage form is administered in a solution or semi-solid state and undergoes a phase transition to a solid or semi-solid state at the injection site. This transition is driven by external environmental changes, such as variations in temperature, ionic strength, pH, or light, thereby establishing a localized drug depot [4]. At the site of administration, in situ gels can rapidly undergo a phase transition, transforming from a liquid state into a non-chemically crosslinked semi-solid dosage form. Prior to administration, they exist as high-molecular-weight polymer solutions loaded with drugs. This enables the controlled and sustained release of the encapsulated drug over time. Notably, injectable in situ gel drug delivery systems have garnered significant interest in recent years for their potential in anti-tumor therapies.

**Table 1:** Published research results on in situ gel injection preparations.

Launch Date	Product Name	Active Ingredient	Specification	Administration Route	Duration of Action
1998	Atridox [5]	Doxycycline Hydrochloride	50 mg	Periodontal drug delivery	7 days
2002	Eligard [6]	Leuprorelin Acetate	7.5 mg~45 mg	Subcutaneous injection	1 month~ 6 months
2018	Perseris [7]	Risperidone	90 mg/0.6 mL; 120 mg/0.8 mL	Subcutaneous injection	1 month
2017	Sublocade [8]	Buprenorphine	100 mg/0.5 m; 300 mg/1.5 mL	Subcutaneous injection in the abdomen	/
2018	Buvidal [9]	Buprenorphine	8 mg, 64 mg, 96 mg, 128 mg, or 160 mg	Subcutaneous injection in the abdomen	7 days
2021	Posimir [10]	Bupivacaine	660 mg/5 mL	Intra-articular administration	72 h
2021	Zynrelef [11]	Bupivacaine/ Meloxicam	28.6 mg/mL Bupivacaine/ 0.86 mg/mL Meloxicam	Drug administration through surgical incision	72 h
2023	Brixadi [12]	Buprenorphine	weekly; 8,162,432 mg monthly; 6,496,128 mg	Subcutaneous injection	7 days or 1 month

Typically, this type of drug delivery system employs polymer materials that respond spontaneously to physiological stimuli. Before administration, the system exists in a low-viscosity solution state. This gel acts as a localized drug depot, facilitating the sustained release of anti-tumor agents, thereby enhancing the therapeutic efficacy and achieving tumor destruction to a certain extent. Studies have shown that condensates formed by phase separation can concentrate certain anticancer drugs, such as cisplatin and tamoxifen, both in vitro and in tumor cells. This concentration effect may enhance the pharmacodynamics of the drugs, that is, their efficacy in the body. Based on this phenomenon, researchers have proposed a new drug design approach: developing small-molecule drugs that can target specific phase separation condensates. This design may improve the specificity and efficacy of the drugs [13]. The ability of phase separation condensates to concentrate drugs is similar to the function of in situ gels in drug delivery. Therefore, the design of small-molecule drugs targeting specific condensates is closely related to the targeted release of drugs using in situ gels.

**Figure 1:**
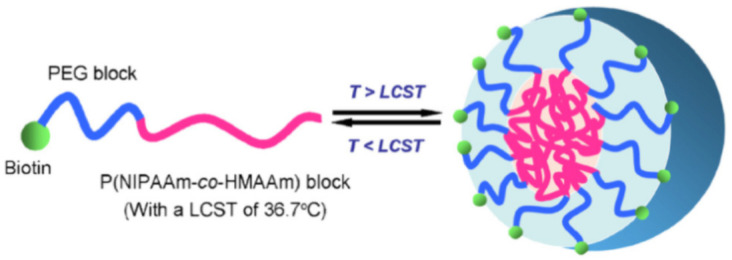
Schematic illustration of the thermally induced structural change in Biotin-PEG-b-P(NIPAAm-co-HMAAm) micelle [15].

Similarly, poly(N-isopropylacrylamide) (PNIPAAm) is a thermoresponsive polymer with a lower critical solution temperature (LCST) of approximately 33 °C. This LCST can be adjusted upward or downward by incorporating various hydrophilic or hydrophobic components. As a result, numerous polymer materials based on PNIPAAm have been developed for use as drug carriers in temperature-responsive gels [14]. Zhang and colleagues designed a biotin-polyethylene glycol-b-P(NIPAAm-co-HMAAm) triblock copolymer. Above its LCST, this polymer spontaneously self-assembles into micelles with a P(NIPAAm-co-HMAAm) core surrounded by a polyethylene glycol (PEG) outer layer (Figure 1) [15]. These biotin-modified micelles can bind to various ligands, enabling targeted drug delivery. This versatility makes them highly promising for tumor-targeted chemotherapy applications [16].

Broadly, in situ gel systems can be categorized into two types based on their mechanisms of gelation. The first type relies on stimuli-responsive phase transitions triggered by the physiological environment. The second type involves gelation induced by external irradiation, such as ultraviolet (UV) or visible light, which further expands the versatility of these systems for site-specific and controlled drug delivery applications [17]. For instance, temperature-sensitive in situ gels represent a significant advancement over traditional gel formulations. At ambient room temperature, these gels exist as uniform and semi-transparent liquids. However, they possess the remarkable ability to undergo reversible phase transitions in response to temperature variations. This thermosensitivity allows them to be mixed with therapeutic agents, such as cells, drugs, and growth factors, and to be subsequently administered in a solution state at lower temperatures. Upon exposure to physiological body temperature, these solutions rapidly transition into a gel state. This phase change results in the formation of a gel reservoir at the site of administration, facilitating the localized and sustained release of the incorporated drugs. This characteristic makes temperature-sensitive in situ gels particularly valuable for applications in regenerative medicine and targeted drug delivery, where precise control over drug release and retention at the injury site is crucial [18].

To better understand the current state and emerging trends of in situ gel drug delivery research, bibliometric analysis serves as an indispensable tool. Bibliometric analysis employs unstructured data derived from a vast body of published studies to comprehensively describe, analyze, and visualize the scientific knowledge landscape of a particular field. This method has been extensively utilized to conduct systematic reviews of the existing literature, offering valuable insights into the knowledge structure, development trajectories, and research hotspots within a specific area of interest. By applying bibliometric analysis to the field of in situ gel drug delivery systems, researchers can gain a clearer understanding of its evolution, identify key contributors, and uncover potential opportunities for further exploration and innovation [19]. Advanced tools like VOSviewer, CiteSpace, Bicomb, and BibExcel are widely used for bibliometric analysis and visualizing research trends. These tools systematically analyze large volumes of scholarly data, offering insights into technological and scientific advancements within a field. For example, VOSviewer constructs bibliometric networks (e.g., co-authorship, keyword co-occurrence, and citation relationships) to identify influential authors, institutions, and journals. CiteSpace detects emerging trends and research frontiers through citation bursts and co-citation networks, revealing the evolution of a field. Bicomb focuses on keyword extraction and co-occurrence analysis, while BibExcel provides flexible tools for data preprocessing and analysis.

By utilizing these tools, researchers can assess a discipline’s technological maturity, knowledge structure, research hotspots, and gaps, identifying core topics and emerging areas for further exploration. Applied to in situ gel drug delivery systems, these methods offer a macroscopic view of the field’s development, highlight influential studies and contributors, and map the trajectory of ongoing advancements [20]. Bibliometric analysis serves as a powerful methodological approach that utilizes unstructured data derived from a substantial body of scholarly publications to systematically describe, quantify, and map the progression of scientific knowledge. By analyzing patterns in authorship, citation networks, keyword co-occurrences, and thematic trends, bibliometric studies provide a macroscopic overview of the intellectual structure and developmental trajectory of a research field.

By employing bibliometric tools and techniques, researchers can not only assess the historical and current scope of a topic but also project future trends and emerging directions. This makes bibliometric analysis an indispensable tool for strategic research planning, thus fostering interdisciplinary collaboration and promoting innovation. In the context of in situ gel drug delivery systems, such an approach can illuminate research hotspots, highlight evolving trends, and provide actionable insights into how this novel drug delivery platform can achieve greater clinical relevance and impact [21]. This analysis is instrumental in evaluating research achievements, identifying prolific authors, institutions, and contributing journals, and elucidating the dissemination of knowledge across specific subject areas [22]. Notably, bibliometric analysis focused on in situ gel drug delivery systems remains relatively sparse. In light of this, our study sought to systematically examine the prevailing research trends, publication volumes, citation frequencies, and critical keywords associated with in situ gel drug delivery systems over the past two decades using data sourced from the Web of Science Core Collection database.

In situ gel drug delivery systems are a pioneering method in pharmaceutical sciences, but this area continues to develop, with many uncharted paths and unrealized opportunities. Our research not only outlines the current state of the field but also highlights critical areas of focus, including the creation of responsive gels to stimuli, the incorporation of nanotechnology, and the investigation of new biocompatible substances. By utilizing sophisticated bibliometric tools like VOSviewer, we successfully simplified the complexities found in the literature, offering valuable perspectives on the present research status and pinpointing possible future avenues for exploration and advancement within this domain. In a prior study, Fatimah et al. [23] performed a bibliometric analysis of articles on nanoemulsions and/or in situ gels for ocular drug delivery systems using the Scopus database and covering publications from 2011 to 2021. Building upon their foundational work, our research significantly advances the field by expanding both the scope and depth of investigation. Specifically, we broadened the research focus to encompass a wider range of in situ gel drug delivery systems, beyond the limited context of ocular applications. Furthermore, we extended the temporal scope to capture more recent developments, ensuring a comprehensive and up-to-date analysis. Additionally, by concentrating on the Web of Science (WOS) database, we leveraged a more authoritative and widely recognized source, enhancing the reliability and academic impact of our findings. These methodological improvements position our study as a more robust and inclusive contribution to the field of in situ gel drug delivery systems.

In conclusion, our bibliometric evaluation not only fills an important void in the existing literature but also emphasizes the innovative and transformative nature of in situ gel drug delivery systems. By systematically analyzing the research terrain and forecasting upcoming trends, we establish a basis for strategic research design and cross-disciplinary teamwork, ultimately propelling the field toward heightened clinical significance and broader societal influence.

## 2. Methods

### 2.1. Data Sources and Search Strategies

**Figure 2:**
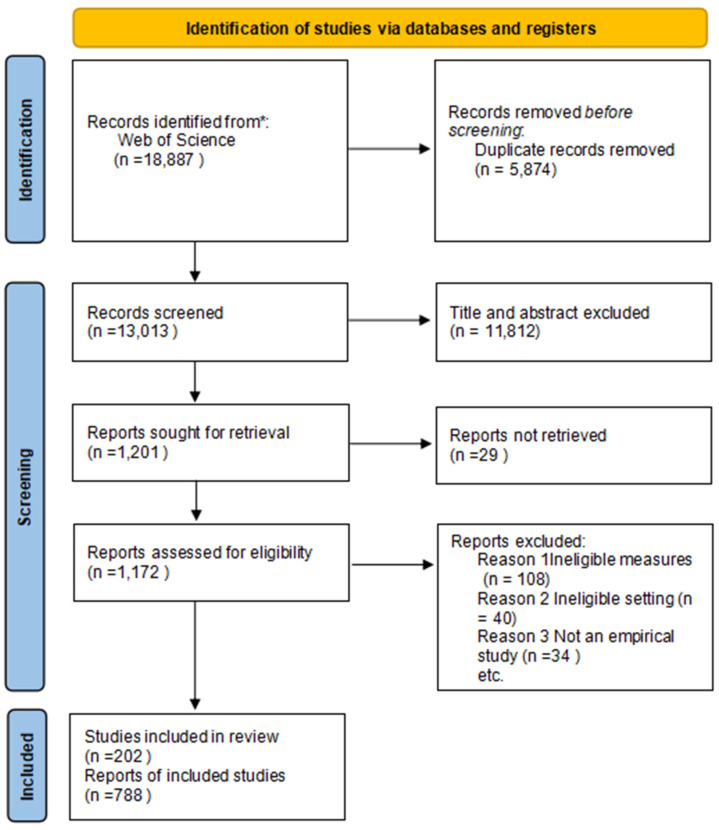
PRISMA flow diagram of the steps of article search. The asterisk (*) indicates that the literature search was conducted exclusively in the Web of Science Core Collection database, from which 18,887 records were initially identified.

A detailed literature search was conducted following the guidelines of the Preferred Reporting Items for Systematic Reviews and Meta-Analyses (PRISMA) 2020 Checklist (Table S1) and the PRISMA 2020 flow diagram (Figure 2) [24].

The inclusion criteria for this study were meticulously defined to ensure a focused and relevant dataset. The selected topic was in situ gel drug delivery systems, with the article types restricted to research papers or reviews and the language specified as English. The exclusion criteria encompassed letters, conference abstracts, editorial materials, book chapters, and other article types that did not align with this study’s objectives. 

The Web of Science Core Collection database served as the primary source for data retrieval, with the “Science Citation Index Expanded (SCI-EXPANDED)” chosen as the citation index. The time span for the analysis was established from 1 January 2003 to 31 December 2023, ensuring a comprehensive overview of two decades of research. The publication language was confined to “English,” and document types were limited to “article” and “review article.”

For author analysis, a threshold of a minimum of five publications was set to ensure the prominence and influence of authors in the field. Similarly, institutional analysis required a minimum of five publications to identify leading research entities. In terms of citation and co-citation analysis, a minimum citation frequency of five was established to highlight significant works and their interconnections within the scientific community. This rigorous methodology enabled a robust and insightful analysis of the research landscape concerning in situ gel drug delivery systems.

### 2.2. Bibliometric Analysis

In this study, we used VOSviewer (version 1.6.17) to analyze CSV export results, examining institutions, sources, authors, papers, co-citations, and keyword co-occurrence. Data were cleaned using an Excel thesaurus program to ensure accuracy and remove duplicates. VOSviewer facilitated network exploration and graph readability by setting thresholds for publications and citations, dividing the network into color-coded clusters for thematic clarity. Node sizes reflected importance, with larger nodes indicating higher influence.

Complementing VOSviewer, CiteSpace (version 6.1.R3) was employed to visualize research frontiers, knowledge bases, and key literature, identifying critical junctures and trends in the field. This dual-software approach provided a comprehensive bibliometric analysis, offering valuable insights into research on in situ gel drug delivery systems [25]. 

## 3. Results

### 3.1. Data Searches

In this study, we retrieved 1172 articles related to in situ gel drug delivery systems. After screening titles and abstracts, 182 irrelevant articles were excluded, leaving 990 articles for analysis. This study covered publications from 1 January 2003 to 31 December 2023. Each article was downloaded, with uncertain ones further evaluated. The search was completed in one day to avoid errors from database updates. Two separate reviewers evaluated the titles and abstracts to determine their relevance. Full-text articles were reviewed for eligibility according to the predefined inclusion and exclusion criteria. Any disagreements that arose were addressed through discussion or, if necessary, by seeking input from a third reviewer.

The Web of Science (WOS) database was used for article retrieval and classification, while Excel facilitated statistical analysis of publication trends, core authors, countries, institutions, journals, and keywords. For visual analysis, CiteSpace 6.2.R4 (version 6.4 R1, 64-bit) was employed to explore research trends and intellectual structures. Articles were imported into CiteSpace in .txt format, deduplicated, and stored in designated folders for analysis [19,20,26]. This combined approach ensured a comprehensive understanding of the field’s development [27].

### 3.2. Analysis of Document Output and Citation Frequency

**Figure 3:**
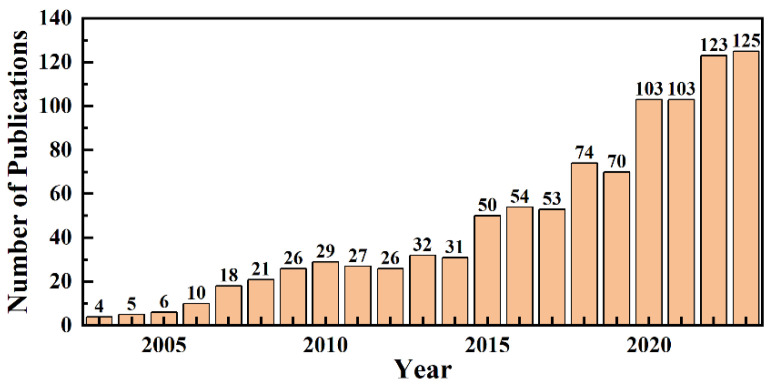
Number of publications of in situ gel applications from 2003 to 2023.

Since 2003, research on in situ gel drug delivery systems has shown a general upward trend with periodic fluctuations, as seen in Figure 3. Data from the WOS database, filtered for relevance, indicate a temporary decline in publications in 2014, followed by a recovery in 2015. From 2020 to 2023, publication numbers stabilized at a high level, averaging 113.5 papers annually, peaking at 125 in 2023. Concurrently, citation frequency also rose, reaching a high of 141 citations in 2023. These trends highlight the growing academic interest and significance of in situ gel drug delivery systems in recent years.

### 3.3. Analysis of Authors

**Figure 4:**
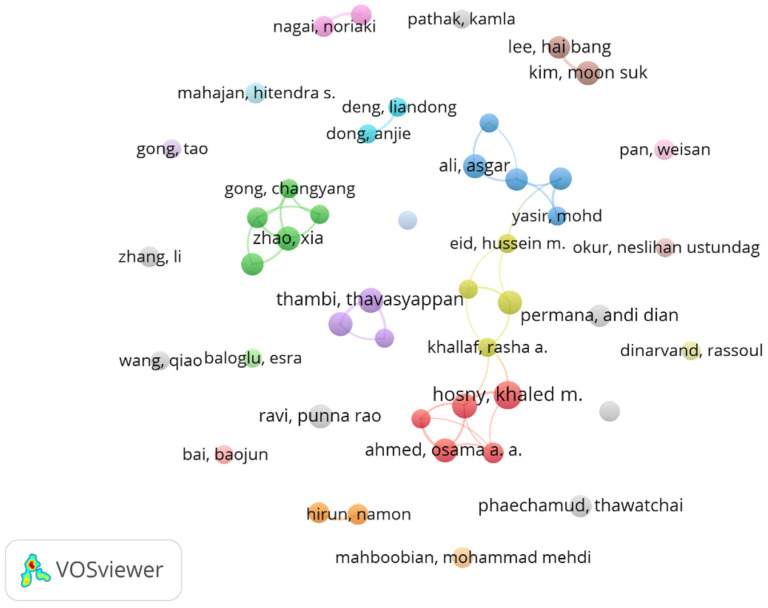
Clustering of the analysis of authors.

The distribution of authorship within the field of in situ gel drug delivery systems reveals key contributors driving research advancements. As shown in Figure 4, among these, Hosny, Khaled M. emerges as the most prolific author, with 11 publications, followed by Alhakamy, Nabil A. andThambi, Thavasyappan, each contributing 9 publications. Additionally, seven authors share a notable publication count of eight papers each:Ahmed, Osama A. A., Ali, Asgar, Kim, Moon Suk, Lee, Doo Sung, Ravi, Punna Rao, Salem, Heba F., and Zhao, Xia. When utilizing VOS Viewer with a minimum publication threshold of five, a total of 46 authors emerged as key contributors, as illustrated in Figure 2. Through co-authorship analysis, eight clusters were initially identified. After excluding outliers, the network was refined to three primary clusters, reflecting strong collaborative dynamics within the field.

Citation metrics further highlight influential contributions. The top five most cited authors are Zhao, Xia (1233 citations), Gong, Changyang (1016 citations), Qian, Zhiyong (984 citations), Shi, Shuai (765 citations), and Pan, Weisan (589 citations). This indicates that these authors have had significant intellectual influence on the trajectory of research in this domain. A comprehensive analysis underscores the prominence of certain authors whose work aligns with high productivity, extensive collaborations, and substantial citation impact. Notably, Zhao, Xia, Hosny, Khaled M., and Kim, Moon Suk stand out as leading figures exemplifying these attributes.

The primary research directions in the field are diverse, yet, interrelated. Key areas include the development of biodegradable in situ gel-forming controlled drug delivery systems based on thermosensitive PEG-PCL-PEG hydrogels, the preparation and optimization of drug delivery materials tailored for in situ gel systems, and in vivo studies of injectable in situ gel drug delivery systems for clinical applications. These research themes reflect the ongoing focus on advancing material science, optimizing therapeutic efficacy, and translating innovative drug delivery technologies into practical medical solutions.

### 3.4. Analysis of Publishing Countries and Institutions

The geographical and institutional analysis of research on in situ gel drug delivery systems highlights notable patterns in productivity, collaboration, and citation impact. South Korea leads with an average of 41 citations per article, followed by the United States (38 citations), China (32 citations), India (27 citations), Egypt (27 citations), and Saudi Arabia (20 citations). Despite ranking third in citation frequency, China is the global leader in research output, with 261 published articles, reflecting its significant contribution to the field.

**Figure 5:**
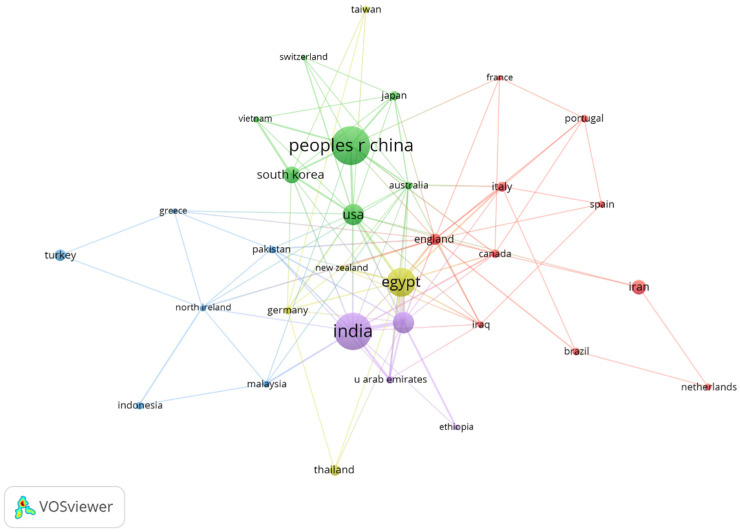
Clustering of the analysis of countries.

A co-authorship network analysis, with a minimum publication threshold of five papers, reveals a collaborative network of 33 nodes, forming five distinct clusters after removing isolated nodes. These clusters, depicted in Figure 5, represent collaborative relationships among institutions, with China at the central position, indicating its key role in fostering international research collaboration. The node size reflects each institution’s total association strength, highlighting their importance in global research partnerships.

In terms of institutional contributions, the top five institutions by published articles are Cairo University (34 articles), King Abdulaziz University (34 articles), Sichuan University (26 articles), Shenyang Pharmaceutical University (25 articles), and Al-Azhar University (17 articles). Cairo University leads not only in output but also in its focus on gel-targeted drug delivery research. Sichuan University stands out with the highest average citation frequency of 70 citations per article, excelling in research on thermosensitive in situ gel drug delivery systems and environmentally sensitive hydrogels.

Considering total association strength as an indicator of institutional importance, the top five institutions are King Abdulaziz University, Beni Suef University, Cairo University, Prince Sattam Bin Abdulaziz University, and King Saud University. These institutions demonstrate strong collaborative networks and play significant roles in advancing global research efforts.

Overall, this analysis underscores the dynamic interplay between institutions and countries in driving innovation within the field of in situ gel drug delivery systems. China’s strong centrality and the high citation impact of institutions like Sichuan University highlight the growing prominence and internationalization of research in this domain.

### 3.5. Keyword Analysis

**Figure 6:**
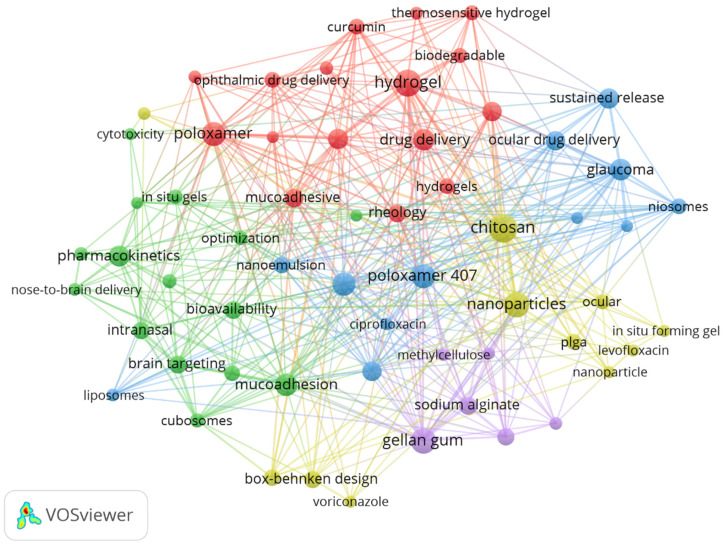
Keyword clustering.

The analysis of keyword frequency within the research on in situ gel drug delivery systems provides valuable insights into prevailing research themes and emerging trends. The top ten most frequently occurring keywords are drug delivery, Vitro release, formulation, nanoparticles, hydrogels, chitosan, optimization, poloxamer, pharmacokinetics, and mucoadhesive, as visually represented in Figure 6. These keywords reflect the core components and considerations inherent in the development and application of in situ gel drug delivery systems.

The prominence of keywords such as drug delivery and Vitro release underscores the fundamental importance of ensuring effective and controlled release mechanisms in drug delivery research. Formulation and optimization highlight the continuous efforts to refine and enhance the design of delivery systems for improved therapeutic outcomes. The inclusion of nanoparticles and hydrogels indicates the integration of advanced materials science in the creation of innovative delivery platforms. 

Chitosan, a natural polymer, and poloxamer, a thermosensitive polymer, are frequently mentioned for their roles in enhancing the biocompatibility and responsiveness of these systems. Pharmacokinetics is crucial because understanding the absorption, distribution, metabolism, and excretion of drugs within these systems is essential for ensuring efficacy and safety. Lastly, mucoadhesive properties are significant for improving the retention and localized delivery of drugs at mucosal surfaces.

The VOSviewer hotspot analysis further reveals that recent research has increasingly focused on the research and optimization of new materials required for the preparation of in situ gel drug delivery systems. This focus reflects a broader trend towards leveraging novel materials to enhance the functionality, stability, and specificity of drug delivery mechanisms. As the field continues to evolve, the convergence of material science and pharmaceutical technology is likely to drive further innovations and breakthroughs in developing sophisticated drug delivery solutions.

#### 3.5.1. Hydrogels

Cluster 1 in the analysis prominently features hydrogels, renowned for their three-dimensional network structures characterized by high water content, which remain stable without decomposing or dissolving, even in excess water. The origins of hydrogel research date back to 1960, with a significant resurgence of interest in recent years, as highlighted by Peppas et al. [28]. The future prospects for hydrogels as drug carriers appear exceedingly promising.

Hydrogels possess a wide array of distinctive properties, making them particularly well-suited as smart carriers within drug delivery systems. These unique characteristics underscore their remarkable potential and versatility. Over the years, substantial innovations have been introduced, significantly advancing the hydrogel domain and expanding their practical applications.

Foreseeing future research directions, it is imperative to address the specific demands associated with the development of advanced and sophisticated hydrogel-based delivery systems. The crosslinked architectures of polymers, prepolymers, and monomers are anticipated to remain the preferred biomaterials for synthesizing these systems. Molecular simulations present an excellent approach for both microscale and macroscale modeling, offering mechanistic insights into the swelling and deswelling behaviors of hydrogels. This dynamic simulation approach effectively captures the molecular interactions among polymer chains, water or solvent molecules, and the bonds within the polymer structure.

Various molecular models have been employed to characterize both the qualitative and quantitative aspects of swelling and deswelling in polyelectrolyte hydrogels. These insights are instrumental in guiding researchers toward optimizing the properties of hydrogels to meet specific criteria. By focusing on these areas, future studies can enhance the design and functionality of hydrogel-based systems, ensuring they fulfill the requisite standards for advanced applications, as noted by Saunders et al. [29].

#### 3.5.2. Nanoparticles

Cluster 2 primarily emphasizes chitosan, nanoparticles, PLGA (poly(lactic-co-glycolic acid)), and other widely utilized carrier materials in the realm of in situ gel drug delivery systems, with a particular focus on nanogels. Among these, nanogels have garnered considerable attention due to their unique and advantageous properties, including a large surface area and exceptional structural stability. These attributes make nanogels highly suitable for various drug delivery applications.

Nanoparticles, as an emerging class of drug carriers, have demonstrated a multitude of benefits in drug delivery [30]. These include achieving sustained-release effects, extending the duration of drug action, and enabling targeted delivery, all of which significantly enhance therapeutic efficacy [31]. Moreover, nanoparticles contribute to reducing toxic side effects, improving drug stability, and simplifying storage requirements. Importantly, they also open avenues for exploring novel drug administration routes, broadening the scope of pharmaceutical applications.

In recent years, nanogels formed through the self-assembly of hydrophobically modified polysaccharides have received widespread attention and extensive research. This interest is largely attributed to their potential value in drug delivery systems. However, the ability of in situ gels to achieve the intended therapeutic effect is not solely dependent on the physicochemical properties and pharmacological activity of the drug itself. It is also intricately linked to the composition of the formulation, which includes key elements such as the active pharmaceutical ingredient, gel matrix material, absorption enhancers, pH regulators, preservatives, and other excipients [32].

Recent studies have also highlighted the significance of polyethylene glycol (PEG) block copolymers synthesized from polylactic acid and polyethylene glycol analogs as common thermosensitive materials for in situ gel formulations [33]. These copolymers exhibit the ability to respond dynamically to environmental temperature changes, thereby enabling intelligent and controlled drug release. This thermosensitivity enhances the precision and efficacy of drug delivery, underscoring the importance of incorporating such advanced materials into future formulations.

By leveraging these innovative materials and systematically optimizing formulation compositions, researchers can further augment the functionality and therapeutic potential of in situ gel drug delivery systems, paving the way for breakthroughs in pharmaceutical science and technology [34].

#### 3.5.3. Gellan Gum

Cluster 3 highlights key gel matrix materials, such as gellan gum, sodium alginate, and methylcellulose, which are critical in drug formulations. Their properties and dosages significantly influence drug diffusion and release. Gellan gum, a linear polysaccharide composed of glucose, glucuronic acid, and rhamnose in a 2:1:1 ratio, forms a three-dimensional network in ion-rich environments without chemical crosslinking. This enhances its safety, biocompatibility, and applicability in food, medicine, and cosmetics while minimizing toxic side effects.

**Figure 7:**
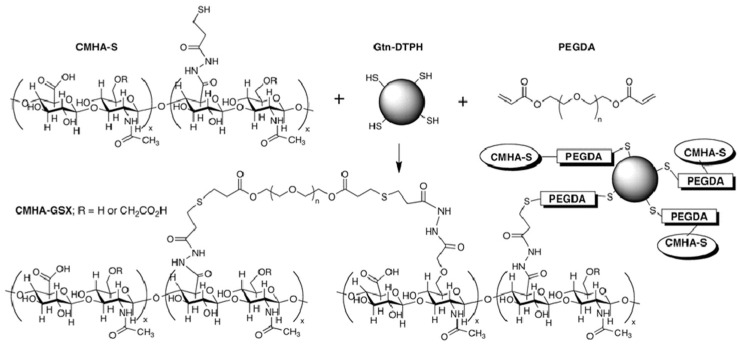
Thioether crosslinked semi-synthetic ECM formed by crosslinking CMHA-S with thiol-modified gelatin using the bifunctional crosslinker PEGDA [37].

Owing to its natural origin, gellan gum exhibits excellent biocompatibility, biodegradability, and injectability, making it a highly promising material for injectable gel applications. Similarly, thiol-modified carboxymethyl hyaluronic acid (CMHA-S) and thiol-modified gelatin (Gtn-DTPH), when crosslinked with the bifunctional agent poly (ethylene glycol) diacrylate (PEGDA), can form biocompatible structures in the presence of cells or tissues (see Figure 7). Based on this principle, scientists have developed an injectable in situ crosslinked synthetic extracellular matrix (sECM) [35,36].

However, it faces practical limitations. Its gelation temperature exceeds body temperature, and the process occurs almost instantaneously, leaving little room for adjustment. Additionally, in vivo, divalent cations in the gel may be replaced by monovalent cations, compromising the gel’s structural integrity. To address these limitations, the chemical modification of gellan gum has become a prominent area of research. Researchers are exploring various modification strategies to enhance the material’s performance, enabling more precise control over gelation properties and improving its suitability for biomedical applications. By advancing these modifications, gellan gum can be optimized for a wider range of therapeutic applications, maintaining its advantageous properties while overcoming inherent constraints [38]. For instance, Miyamoto et al. [39] successfully synthesized carboxymethylated xanthan gum with improved water solubility by introducing sodium chloroacetate into an alkaline xanthan gum solution. The carboxymethylation process significantly modified the solubility and gelation properties of xanthan gum, demonstrating its potential for enhanced application versatility. Notably, at 25 °C, the concentration of the modified xanthan gum could reach as high as 10% while maintaining its sol state, even when cooled to 0 °C. This alteration in behavior underscores how chemical modification can fundamentally enhance the functional properties of natural polymers, paving the way for innovative applications in pharmaceutical formulations and other fields that require precise control of gelation and solubility characteristics.

#### 3.5.4. Poloxamer

Cluster 4 highlights the pivotal role of poloxamer 407, particularly in research on its application for sustained drug release and the clinical management of glaucoma. Poloxamer 407, commercially recognized as pluronic F127, is a triblock copolymer with a molecular weight of approximately 12.6 kDa (POE101–POP56–POE101). Its molecular structure consists of a central hydrophobic block of polyoxypropylene (POP) flanked by two hydrophilic blocks of polyoxyethylene (POE), with polyoxyethylene constituting roughly 70% of the molecule. This high proportion of hydrophilic units significantly enhances its solubility in aqueous environments, making it an exceptional candidate for biopharmaceutical applications [40]. A notable property of poloxamer 407 is its thermo-reversible behavior, which has positioned it as a critical component in mucosal drug delivery systems. Specifically, its temperature-sensitive sol-to-gel transition is especially advantageous for applications requiring localized delivery. At lower temperatures, poloxamer 407 exists as a free-flowing solution, facilitating ease of administration. However, when exposed to physiological temperatures (~37 °C), it undergoes gelation, forming a stable and viscous gel. This property allows for prolonged residence time at the site of administration and sustained drug release, reducing dosing frequency and improving therapeutic outcomes [41]. The thermosensitivity of aqueous poloxamer 407 solutions arises from unique molecular interactions [42]. At lower temperatures, the copolymer chains are surrounded by a hydration shell, stabilized by hydrogen bonding between the hydrophilic polyoxyethylene segments and water molecules [43]. As the temperature increases, the hydrogen bonds break down, resulting in the desolvation of the hydrophilic chains. This process enhances hydrophobic interactions among the polyoxypropylene segments, leading to the self-assembly of spherical micelles. These micelles further aggregate into a closely packed structure, ultimately triggering the gelation process. This reversible transition between sol and gel states provides remarkable versatility for various biomedical applications, particularly in mucosal delivery systems where compatibility with sensitive tissues is critical [44].

Moreover, poloxamer 407 exhibits excellent biocompatibility and is considered safe for use as it does not damage mucosal membranes. This feature, combined with its thermo-reversible behavior, positions it as a highly promising material in the development of advanced drug delivery platforms, including those targeting chronic diseases such as glaucoma. Recent studies continue to explore its potential, unveiling novel formulations and expanding its utility across diverse therapeutic areas.

Formulations incorporating poloxamer 407 at concentrations ranging from 15 to 30% w/w demonstrate gelation at physiological temperatures, making them ideal for various biomedical applications. These thermosensitive hydrogels have been employed to deliver active pharmaceutical ingredients with diverse physicochemical properties, facilitating controlled release and enhancing therapeutic efficacy [44].

Poloxamers, particularly poloxamer 407, are extensively utilized as thermogel polymers in the development of effective ophthalmic drug delivery systems. Their favorable attributes, including low toxicity, thermosensitive sol–gel transition, reversible thermogel properties, and efficient sustained-release capabilities, make them suitable candidates for ocular applications. The topical administration of medications is generally preferred for treating eye conditions due to the presence of the blood–aqueous barrier and the blood–retinal barrier, which impede the penetration of systemically administered drugs into ocular tissues [45]. In particular, poloxamers have been successfully employed in in situ gels and other formulation carrier materials. For instance, in situ gels designed for delivering the antifungal agent voriconazole were developed using a combination of poloxamer 407 and carboxymethyl cellulose as the gel matrix. These formulations, when applied to the eye, demonstrated excellent biocompatibility by causing no irritation, thereby underscoring their potential for safe and effective ocular drug delivery [46]. The continued exploration of poloxamer-based formulations holds promise for advancing the field of ophthalmic therapeutics, providing innovative solutions that address both the challenges of drug delivery and patient comfort. As research progresses, these formulations are likely to play an increasingly pivotal role in the management of various eye diseases, optimizing drug bioavailability and patient adherence. 

#### 3.5.5. Pharmacokinetics

Cluster 5 primarily delves into the pre-formulation studies of novel in situ gel formulations, with a particular emphasis on their application in nasal drug delivery systems to achieve brain-targeting effects. The distinctive solution-to-gel transformation property of in situ gels allows them to remain at the site of administration for extended periods, creating a sustained local drug concentration. This characteristic has positioned in situ gels as an advantageous platform for the development of drug delivery systems with enhanced targeting capabilities, especially when combined with active pharmaceutical ingredients (APIs).

The nasal route offers a promising alternative for delivering drugs to the brain, primarily due to its ability to bypass the blood–brain barrier (BBB), a significant obstacle in central nervous system (CNS) drug delivery. The transport of drugs from the nasal cavity to the brain is mediated through a combination of three major pathways: (1) the olfactory nerve pathway, (2) the olfactory mucosal epithelial pathway, and (3) the blood circulation pathway. Among these, the first two pathways are directly associated with the direct absorption of drugs into the brain, while the third pathway involves the systemic absorption of drugs into the bloodstream, after which they must cross the BBB to reach the brain [47].

Notably, the olfactory mucosal epithelial pathway has been identified as the predominant and more rapid route for the absorption of small molecules into the brain compared to the olfactory nerve pathway [48,49,50]. This expedited absorption is attributed to the relatively short distance and high permeability of the nasal mucosa, which facilitates efficient drug transport. By leveraging these pathways, nasal delivery systems can circumvent the limitations imposed by the BBB, thereby achieving direct brain targeting and enhancing the therapeutic potential for the treatment of neurological disorders.

In situ gel formulations, when employed in nasal drug delivery systems, further enhance their efficacy due to their ability to prolong nasal retention, reduce the frequency of administration, and provide sustained drug release. This unique combination of properties not only improves patient compliance but also ensures a high local drug concentration, optimizing drug bioavailability in the brain. As research progresses, this innovative approach is poised to address critical challenges in CNS drug delivery, offering new avenues for the treatment of complex brain diseases with improved precision and therapeutic outcomes.

#### 3.5.6. Stability Analysis of In Situ Gel

The main focus of the provided keywords centers on the stability of in situ gels, a critical factor determining their functionality in drug delivery systems. Gel stability refers to the ability of the gel to maintain its structural integrity, controlled drug release profile, and therapeutic efficacy under physiological conditions. Key parameters for assessing stability include gelation time, mechanical strength, and degradation rate, which are typically evaluated through rheological analysis, in vitro drug release studies, and long-term storage tests. External factors such as temperature, pH levels, and ionic strength can significantly influence gel stability, necessitating careful optimization of polymer composition and crosslinking density. For instance, thermosensitive polymers like poloxamer 407 exhibit reversible gelation behavior [51], while pH-sensitive polymers such as chitosan rely on environmental triggers for gel formation [52]. Recent advancements have focused on enhancing stability by incorporating natural and synthetic polymers and developing hybrid hydrogels with improved mechanical properties and stimuli-responsive capabilities. Ensuring long-term stability is essential for achieving consistent therapeutic outcomes and minimizing adverse effects, making it a central area of research in the development of advanced in situ gel systems.

#### 3.5.7. Discussion

**Figure 8:**
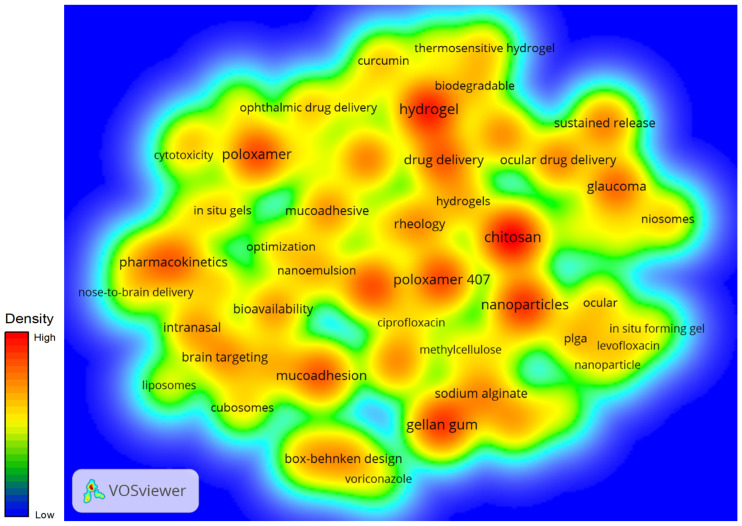
Keyword density.

As illustrated in Figure 8, which provides a keyword density analysis, the progression of hydrogel research over time reveals a distinct evolutionary trend—from bulk gels to microgels and, more recently, to nanogels. This shift highlights the growing sophistication in the design and application of hydrogels, driven by their inherent biocompatibility and their ability to incorporate therapeutic molecules effectively. These properties have catalyzed the development of innovative hydrogel formulations, which are becoming increasingly significant in advancing medical devices and drug delivery systems.

The integration of polymer science into biomedical research has played a pivotal role in the widespread clinical application of hydrogels. Over the past few decades, there has been a marked increase in clinical trials investigating the role of hydrogel formulations in delivering drugs with improved efficacy and precision. A variety of polymers, such as pluronic derivatives, methylcellulose, hydroxypropyl methylcellulose, carboxymethylcellulose, and polyethylene glycols, have been utilized to formulate hydrogel systems with tailored properties [53]. These formulations have demonstrated the capacity to overcome numerous challenges in drug delivery, including enhancing bioavailability, prolonging drug release, and ensuring better patient compliance.

**Figure 9:**
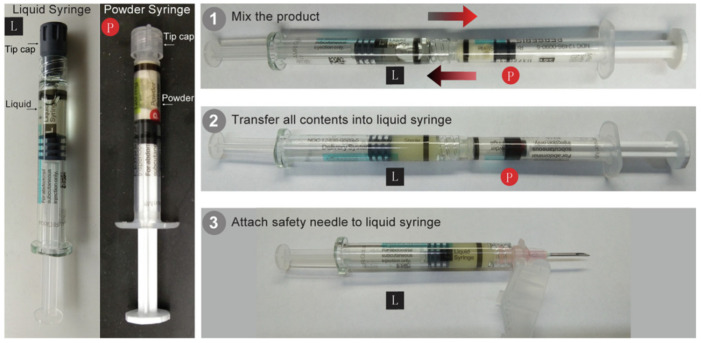
Illustration of in situ gel-forming implant products with a two-syringe mixing system and instructions for use (Perseris^®^) [59].

Various well-established types of in situ gel drug delivery systems, which rely on phase transitions triggered by physical stimulation, have been developed. Examples include Atrigel^®^, SABER^®^, and Fluid Crystal^®^ technologies. These advancements have successfully transitioned from the research phase to clinical use, and numerous products based on these technologies have received marketing approval from regulatory bodies such as the FDA and EMA. Atrigel^®^ technology is categorized under the polymer precipitation gel system, initially developed by Dunn et al. in 1987. As shown in Figure 9, Perseris® is the product currently developed based on this technology and has been approved for marketing by both the FDA and EMA.To date, several products leveraging this technology, have received approval from both the FDA and EMA [54].

SABER^®^ technology, developed by Durect Corporation, is a small molecule-based sustained-release injection system. Posimir^®^, utilizing this platform, is an FDA-approved bupivacaine extended-release solution for postoperative pain management, providing up to 72 h of pain relief and reducing opioid use after arthroscopic surgery. Similarly, Relday^®^, developed by Zogenix, employs SABER^®^ technology to deliver risperidone as a monthly subcutaneous injection for schizophrenia treatment. Phase II trials confirmed its ability to maintain stable therapeutic blood levels, demonstrating its potential for long-term efficacy [55,56,57]. 

Fluid Crystal^®^ technology, created by Camurus, is a liquid crystal gel system. The Fluid Crystal^®^ injection depot consists of a lipid liquid that has the active ingredient dissolved in it and can be administered subcutaneously with a standard syringe equipped with a fine needle. Upon contact with the fluid in the tissue, the lipid solution is converted to a liquid crystal gel, thereby efficiently encapsulating the active ingredient [58].

Recent research has focused on innovatively modifying hydrogel systems for targeted drug delivery, employing advanced nanotechnology methods such as nanoparticles, liposomes, and nanocomposites. These approaches enhance the precision, effectiveness, and therapeutic outcomes of hydrogel-based delivery systems. Hydrogels have become a versatile platform due to their adaptability and compatibility in targeted applications. Current efforts prioritize developing and optimizing new hydrogel materials to improve therapeutic agent stability, bioavailability, and controlled release. By advancing material design and functionalization, researchers aim to expand hydrogels’ clinical applicability, positioning them as a cornerstone of future drug delivery systems with transformative potential for treating complex diseases.

### 3.6. Analysis of Journals

According to the findings, the top five journals with the highest citation frequencies in the field of in situ gel drug delivery systems are the International Journal of Pharmaceutics (3323 citations), the Journal of Drug Delivery Science and Technology (1416 citations), Biomaterials (1302 citations), Drug Delivery (1262 citations), and the European Journal of Pharmaceutical Sciences (974 citations), as illustrated in Table 2. These journals collectively represent key sources of knowledge in this research domain, serving as foundational resources for advancements in the field.

**Figure 10:**
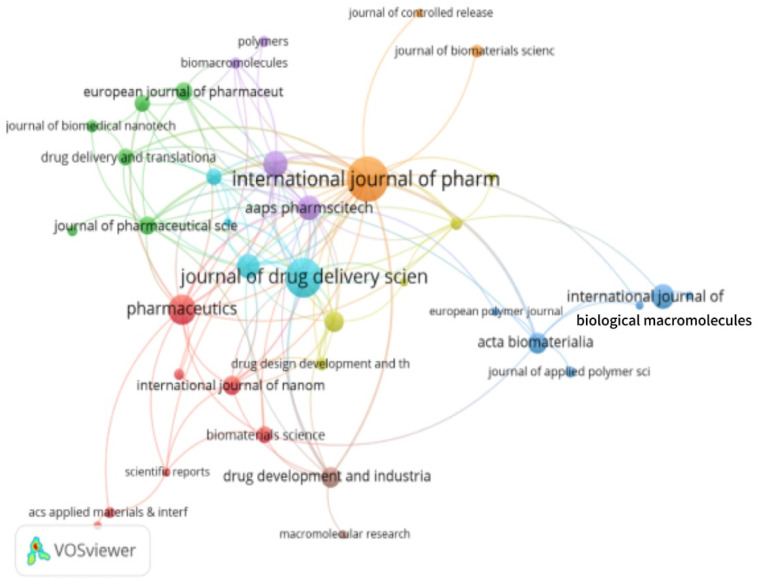
Journal analysis clustering.

Among these, four journals—the International Journal of Pharmaceutics, the Journal of Drug Delivery Science and Technology, Biomaterials, and Drug Delivery—are ranked in the Q1 quartile of the Journal Citation Reports (JCR), underscoring their high impact and authoritative status in their respective disciplines. Notably, the International Journal of Pharmaceutics and the Journal of Drug Delivery Science and Technology are recognized as two of the most influential international journals in pharmaceutical sciences. These journals have played a pivotal role in documenting the historical evolution of in situ gel drug delivery systems, providing researchers with a robust foundation to build upon, as depicted in Figure 10.

In addition, Biomaterials, which is one of the leading journals in the interdisciplinary field of biomaterials science, stands out for offering innovative insights into the selection and development of novel carrier materials. The journal has significantly influenced the preparation of advanced in situ gel formulations by guiding researchers in the design and optimization of cutting-edge materials tailored for drug delivery applications. Its contributions are particularly critical in driving material innovation, which is essential for enhancing the performance and versatility of in situ gel systems.

As shown in Figure 10, these top-cited journals not only reflect the academic community’s focus on in situ gel-based drug delivery systems but also illuminate the progressive trends and research priorities in the field. Their high citation frequencies further emphasize the value of their contributions in shaping the scientific discourse and advancing the development of targeted, sustained, and effective drug delivery platforms.

### 3.7. Analysis of Number of Citations

As detailed in Table 3, the top five most frequently cited references in the field predominantly emphasize the development of novel dosage forms, reflecting the innovative and application-driven nature of research on in situ gel drug delivery systems. These studies collectively illustrate the diverse therapeutic potential and adaptability of in situ gels as advanced drug delivery platforms.

Among these references, two studies focus on the nasal administration of in situ gel formulations for the treatment of Alzheimer’s disease (AD). This research highlights the growing interest in intranasal delivery as a non-invasive route to bypass the blood–brain barrier, enabling the efficient targeting of therapeutic agents to the central nervous system. These studies explore the development of in situ gel systems designed to improve drug retention in the nasal cavity, enhance brain bioavailability, and achieve sustained release, which are critical for treating neurodegenerative conditions such as AD.

Another highly cited study investigates the ocular delivery of nanoemulsion-based in situ gels, emphasizing their potential to address the challenges associated with conventional ophthalmic formulations. By integrating nanoemulsions with in situ gelling systems, this research demonstrates a promising approach to enhance bioavailability, precorneal retention time, and therapeutic efficacy for ophthalmic drugs. This advancement is particularly important for treating chronic and complex ocular diseases, where sustained and localized drug delivery is essential.

In addition, one study delves into the development of multifunctional QCS-ODex hydrogels derived from biopolymers for wound dressing applications. These hydrogels, composed of quaternized chitosan (QCS) and oxidized dextran (ODex), exhibit excellent biocompatibility, antibacterial properties, and wound-healing potential. The multifunctionality of these hydrogels underscores the versatility of in situ gel-based materials in addressing diverse biomedical applications beyond drug delivery, particularly in regenerative medicine.

The fifth reference is a comprehensive review that examines the broad spectrum of applications for hydrogels in contemporary pharmaceutical markets. This review provides an in-depth discussion of various modification techniques—including chemical, physical, and nanotechnology-based approaches—that enhance the precision, stability, and therapeutic efficacy of hydrogels. It serves as a valuable resource for understanding the fundamental principles and emerging strategies in the design of hydrogel-based delivery systems.

Collectively, these frequently cited studies underscore the multifaceted nature of in situ gel drug delivery research, spanning fields such as neurodegenerative diseases, ocular therapy, wound healing, and material innovation. By addressing both fundamental challenges and practical applications, these references have significantly contributed to shaping the research priorities and guiding future advancements in the development of targeted, efficient, and patient-friendly therapeutic platforms.

### 3.8. Citation and Co-Citation Analysis

**Figure 11:**
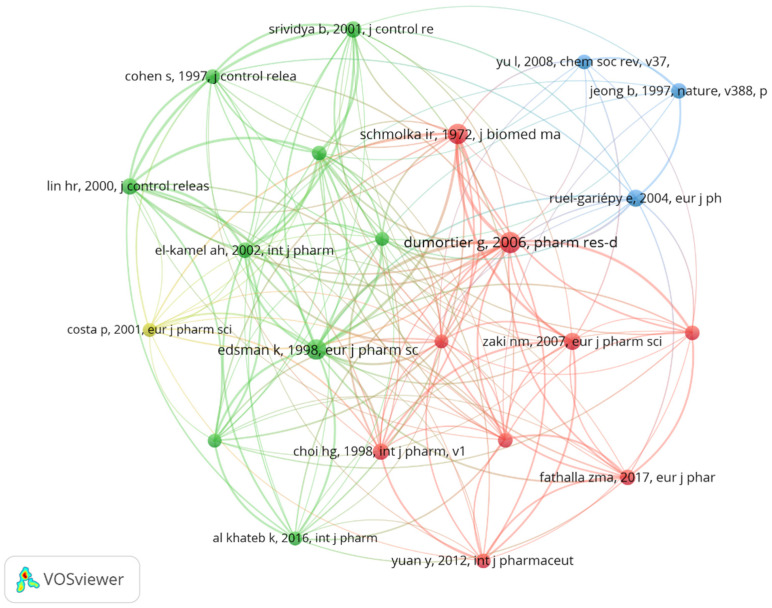
Citation and co-citation analysis clustering I.

**Figure 12:**
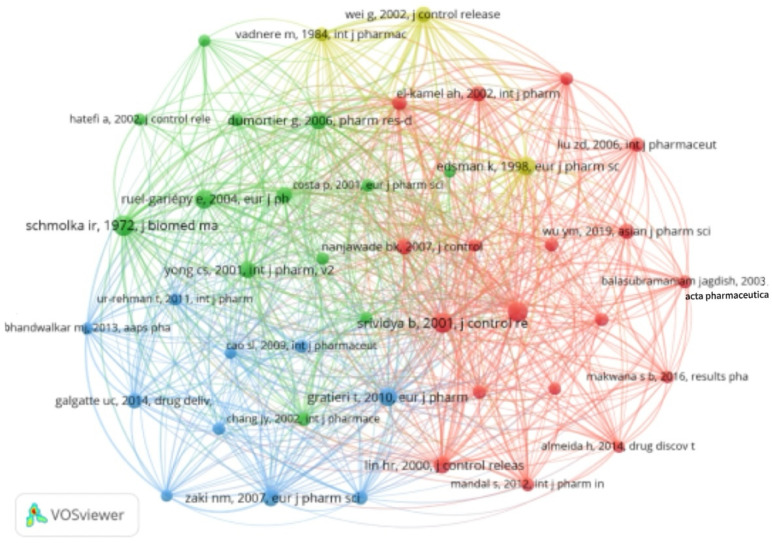
Citation and co-citation analysis clustering II.

The citation and co-citation analysis (Figure 11 and Figure 12) categorized 66 references into four clusters, revealing the interconnected research landscape of in situ gel drug delivery systems. This analysis highlights influential authors and seminal works shaping the field. The top five co-cited authors are Schmolka, Ir. (88 times), Dumortier G. (70 times), Srividya B. (66 times), Ruel-gariepy E. (64 times), and Qi HY. (61 times), reflecting their foundational impact. Notably, Schmolka’s 1972 paper, “Artificial skin. I. Preparation and properties of pluronic F-127 gels for treatment of burns” [60] (Journal of Biomedical Materials Research), introduced innovative methods for developing in situ gels, demonstrating their therapeutic potential for burn wounds. Co-citation analysis emphasizes the role of co-cited studies in identifying academic connections, emerging research directions, and a paper’s influence within the scientific community.

Overall, the insights gained from citation and co-citation analysis provide researchers with a comprehensive understanding of the trends and pivotal contributions within the field of in situ gel drug delivery systems. By tracing the scholarly lineage and recognizing influential works, researchers can build upon established knowledge while pioneering innovative approaches to tackle contemporary challenges in drug delivery and therapeutic development [60]. In situ gel systems for transdermal drug delivery represent a novel advancement, enhancing drug transport efficiency across the skin barrier and addressing challenges like gastrointestinal degradation and hepatic first-pass metabolism. This approach improves drug bioavailability and therapeutic efficacy. The skin’s barrier properties enable sustained or controlled drug release, ensuring stable plasma concentrations, minimizing blood level fluctuations, and reducing adverse reactions. Additionally, the convenience and non-invasive nature of transdermal in situ gels enhance patient adherence.

In parallel, advancements in artificial skin have overcome the limitations of traditional burn treatments, such as autografting, which often cause scarring. Medical gel dressings now prevent and treat hypertrophic scars from burns, trauma, or surgery, offering improved scar management and patient outcomes [61].

In summary, the incorporation of in situ gel technology into transdermal drug delivery and artificial skin development signifies a substantial advancement in medical treatment strategies. This innovation holds the promise of improving therapeutic efficacy, patient comfort, and overall healthcare outcomes. The seminal work by Dumortier, G., titled “A Review of Poloxamer 407 Pharmaceutical and Pharmacological Characteristics”, which was published in 2006, has served as a foundational reference, inspiring numerous subsequent researchers in the exploration and development of poloxamer 407 [62]. This pioneering review has opened new avenues for research, fostering advancements in the application of this versatile polymer in various pharmaceutical contexts. 

However, safety concerns, such as lipid metabolism alterations, renal toxicity, and immunomodulatory or cytotoxic effects, have raised questions about its biocompatibility, particularly for parenteral use [63]. Despite these challenges, poloxamer 407 remains a highly favorable material for temperature-sensitive in situ gels, especially in injectable formulations [64]. Its straightforward preparation, ease of administration, water solubility, and stability make it an ideal platform for the controlled release of macromolecular therapeutics (e.g., proteins and peptides) and bioactive agents (e.g., vaccines and antibodies). Its ability to modulate drug release kinetics and sustain therapeutic effects positions it as a transformative option for advanced injectable drug delivery systems.

**Table 3:** The five most frequently cited references in the literature on in situ gel applications.

	Title	First Author	Source	Year Published	Research Contents	Number of Times Cited
1	Intranasal delivery of Huperzine A to the brain using lactoferrin-conjugated N-trimethylated chitosan surface-modified PLGA nanoparticles for treatment of Alzheimer’s disease	Meng, Q Q [65]	International Journal of Nanomedicine	2018	To develop Huperzine A (HupA)-loaded, mucoadhesive, and targeted polylactide-co-glycoside (PLGA) nanoparticles (NPs) with surface modification by lactoferrin (Lf)-conjugated N-trimethylated chitosan (TMC) (HupA Lf-TMC NPs) for efficient intranasal delivery of HupA to the brain for AD treatment.	234
2	Nanoemulsion-based electrolyte triggered in situ gel for ocular delivery of acetazolamide	Morsi, N [66]	European Journal of Pharmaceutical Sciences	2017	A novel ion-induced in situ nanogel loaded with the anti-glaucoma drug acetazolamide for ocular delivery was developed, aiming to improve the therapeutic effect through sustained drug release.	151
3	Biomedical applications of hydrogels in drug delivery system: An update	Kesharwani, P [67]	Journal of Drug Delivery Science and Technology	2021	This text thoroughly discusses the various applications of hydrogels across contemporary markets and explores the different modification techniques that can be employed to facilitate the precise delivery of therapeutic agents.	130
4	Development, characterization and application of in situ gel systems for intranasal delivery of tacrine	Qian, S [68]	International Journal of Pharmaceutics	2014	This investigation devised an in situ gel formulation for the intranasal delivery of tacrine (THA), an anti-Alzheimer’s disease drug.	102
5	Injectable, self-healing, transparent, and antibacterial hydrogels based on chitosan and dextran for wound dressings	Nie, L [69]	International Journal of Biological Macromolecules	2023	The study developed a versatile QCS-ODex hydrogel that exhibits multiple therapeutic properties, providing a new direction for research on biopolymer-based wound dressings.	47

Overall, in situ gel drug delivery systems hold substantial promise for development and continue to attract considerable research interest across various fields. Future research endeavors should focus on addressing the specific requirements necessary to develop complex and advanced hydrogel-based delivery systems [67]. A critical area of focus should be the optimal selection of biomaterials for synthesizing these systems, with particular attention to the crosslinking structures of polymers, prepolymers, and monomers, as highlighted by Kesharwani et al. [67]. 

## 4. Discussion

This study analyzed studies from 2003 to 2023 on in situ gel drug delivery systems using bibliometric methods and VOSviewer. It identified key contributors, research hotspots, and emerging trends. In situ gels, administered as liquids and undergoing stimuli-responsive gelation under physiological conditions, offer a transformative drug delivery platform. Polymers in these systems respond to environmental changes (e.g., pH, temperature, light, and ion concentration), enabling prolonged drug retention and sustained release, which enhances bioavailability and therapeutic efficacy.

Bibliometric analysis highlighted recurring keywords like hydrogels, poloxamer, nanoparticles, chitosan, and pharmacokinetics, with hydrogels being a central focus due to their water retention, biocompatibility, and mild processing. Hydrogels’ tunable swelling behavior allows precise control over drug release, while their flexibility supports applications in bone and cartilage regeneration, drug delivery, tissue engineering, biosensors, soft robotics, wound healing, and inflammatory treatment. Their ability to mimic the extracellular matrix and respond to stimuli makes them ideal for advanced therapeutic systems.

In conclusion, the insights gained from this bibliometric study highlight not only the rapid advancements in in situ gel drug delivery systems but also the pivotal role of hydrogels and other advanced biomaterials in driving innovation in this field. As research in this area continues to evolve, the integration of novel materials, technologies, and interdisciplinary approaches is expected to further refine these systems, addressing challenges in drug delivery and opening new therapeutic possibilities for a variety of medical applications [70]. Hydrogels are predominantly synthesized from polymers derived from natural, semi-synthetic, and synthetic origins, each offering unique advantages for biomedical applications. Natural polymers, such as polysaccharides, guar gum, collagen, proteins, gum arabic, and nucleic acids, are widely utilized due to their inherent safety, biocompatibility, and adaptability in hydrogel formulation. These naturally sourced polymers exhibit a wide range of desirable properties, including exceptional mechanical stability, biodegradability, and high biocompatibility, making them particularly suitable for advanced biomedical applications. Additionally, their ability to support targeted and specific drug delivery systems further underscores their significance in modern pharmaceutical development [71]. Among the various hydrogel-forming materials, poloxamer 407 has garnered significant attention for its versatile applications as a thermosensitive polymer. Poloxamer 407-based hydrogels are notable for their ability to transition from a liquid state at low temperatures to a gel state at physiological temperatures, facilitating ease of administration and prolonged retention at the site of application. These hydrogels have been employed both independently and in combination with other components as delivery vehicles for therapeutic proteins, such as interleukins, insulin, and bovine serum albumin. This strategy has been shown to effectively extend the half-lives of these proteins, thereby enhancing their therapeutic efficacy and reducing the frequency of administration [72]. The tunable properties of poloxamer-based hydrogels, including their sol–gel transition behavior, biocompatibility, and physicochemical stability, have further solidified their role in drug delivery and biomaterial design.

The versatility of hydrogels extends beyond traditional pharmaceutical applications and into interdisciplinary domains, including tissue engineering, wound healing, biosensing, and even textile-based biomedical innovations. For instance, integrating hydrogels into textile materials has opened new frontiers in wearable biomedical devices and drug delivery platforms. These textile-based hydrogel systems offer unique advantages, such as flexibility, adaptability to various anatomical structures, and the potential for localized and sustained therapeutic delivery [73]. Such applications exemplify the diverse potential of hydrogel-based systems in addressing complex biomedical challenges.

In summary, natural-source polymers have transformed hydrogel synthesis, enabling innovative drug delivery systems. Optimizing materials like poloxamer 407 is expected to advance patient-specific therapies. Early in situ gel research focused on limited natural polymers, often overlooking safety and biocompatibility, which later drove the exploration of biocompatible and biodegradable materials.

Recently, both natural (chitosan, alginate, and hyaluronan) and synthetic polymers (PVA and PEG) have been used for hydrogel-based drug delivery. Natural polymers offer biocompatibility, biodegradability, and extracellular matrix mimicry, with chitosan providing antimicrobial and mucoadhesive properties and alginate enabling gentle encapsulation of sensitive agents. Synthetic polymers like PVA and PEG provide tunable properties, mechanical strength, and enhanced drug solubility.

Combining natural and synthetic polymers overcomes individual limitations, creating hybrid hydrogels with improved mechanical strength, controlled degradation, and stimuli-responsive behaviors. This has led to advanced in situ gels capable of delivering diverse therapeutics (small molecules, proteins, and nucleic acids) with enhanced safety, efficacy, and patient compliance.

Citation analysis shows that nasal–brain drug delivery for Alzheimer’s treatment is emerging as a mature research focus. Researchers are increasingly developing multi-component in situ gel systems, with transdermal, ophthalmic, and injectable preparations also gaining attention. Clinical studies should prioritize multi-center, large-sample, and high-quality research on these topics.

This study highlights recent advances in in situ gels, including hydrogel-based drug delivery via oral [74], rectal [75], vaginal [76], local [77], and ocular [78] routes, and explores their biomedical applications and commercial potential through clinical trials. Future research should focus on developing advanced hydrogel systems using crosslinked polymers, prepolymers, and monomers to meet specific needs.

Injectable in situ gel depot systems (IISGDSs) are gaining attention for their biocompatibility, biodegradability, ease of manufacturing, and ability to protect sensitive biological products. They enable prolonged localized drug release, reducing administration frequency and systemic exposure while improving patient compliance. Their liquid-to-gel transition upon injection ensures precise drug localization, making them ideal for chronic disease management, cancer therapy, and regenerative medicine.

Gellan gum, a natural polymer, is notable for its biocompatibility, biodegradability, and ability to form stable hydrogels under physiological conditions. Its versatility in delivering small molecules, proteins, peptides, and nucleic acids makes it a key material for next-generation IISGDSs, aligning with the demand for safer and sustainable biomaterials. Gellan gum is of great interest for optimizing IISGDS applications in preclinical and clinical research. In conclusion, the rise of IISGDSs reflects a shift toward patient-centric drug delivery platforms that reduce dosing frequency, improve safety, and enhance outcomes. The use of natural polymers like gellan gum underscores the field’s commitment to biocompatible materials. Future advancements in material science and interdisciplinary collaboration will be crucial to fully realizing the potential of IISGDSs and translating them into clinical solutions [79].

With the development of technology, micro-robots have gradually come into people’s view. Their contributions to the biomedical field make it impossible to ignore their broad prospects [80,81,82,83].

Zhong et al. [84] developed a flexible miniature robot based on alginate saline gel, capable of spontaneous deformation in response to environmental ion concentration, achieving multimodal movement. By designing oscillating and rotating magnetic fields, the robot can achieve forward motion in different forms, with a maximum deformation angle of 180°. This highly adaptable robot has potential applications in targeted medical treatments within the digestive system.

## 5. Conclusions

Despite significant advancements in enhancing biocompatibility, drug-loading capacity, drug release mechanisms, and treatment duration, in situ gel drug delivery systems still encounter formidable challenges in clinical applications. Moreover, these systems must exhibit appropriate degradation properties, necessitating further in-depth research on their impact on the adhesion and functionalization of encapsulated adherent cells.

To guarantee the safety and efficacy of in situ gel drug delivery systems, it is crucial to meticulously address concerns related to material selection and quality evaluation. By enhancing experimental methods and implementing stringent quality control measures, the progression and investigation of these systems can be significantly advanced. Such dedicated efforts will play a pivotal role in facilitating the translation of these cutting-edge drug delivery platforms into clinical practice, thereby enhancing therapeutic outcomes and patient care.

Nonetheless, it is important to acknowledge certain limitations of this study. First, as the analysis was solely based on the literature retrieved from the Web of Science database, the findings may not comprehensively represent all research achievements within this field. Second, the relatively limited number of included studies could introduce potential biases, thereby influencing the generalizability of the results. Furthermore, this study did not delve into an analysis of the journals in which the articles were published, which could have provided additional insights into the dissemination trends of this research area.

Future investigations should consider a more extensive and inclusive approach by incorporating multiple databases and expanding the scope of analysis. Additionally, a detailed examination of the publishing journals could further enhance the understanding of the academic impact and dissemination patterns of in situ gel drug delivery systems. Such efforts would contribute to a more holistic and nuanced evaluation of the advancements and applications of these innovative systems across various disciplines.

## Figures and Tables

**Table 2:** The top five journals with the highest citation frequencies.

	Source	Documents	Citations	Impact Factor	JCR
1	International Journal of Pharmaceutics	72	3323	5.3	Q1
2	Journal of Drug Delivery Science and Technology	68	1416	4.5	Q1
3	Biomaterials	11	1302	12.8	Q1
4	Drug Delivery	34	1262	6.5	Q1
5	European Journal of Pharmaceutical Sciences	20	974	4.3	Q2

## Data Availability

The data supporting the findings of this study are available from the Web of Science database but restrictions apply to the availability of these data, which were used under license for the current study, and so are not publicly available. Data are however available from the authors upon reasonable request and with permission of the Web of Science.

## Supplementary Materials

The following supporting information can be downloaded at: https://www.mdpi.com/article/10.3390/pharmaceutics17040451/s1, Table S1: PRISMA Checklist.
